# Characterization of the Cultivable Microbiota in Fresh and Stored Mature Human Breast Milk

**DOI:** 10.3389/fmicb.2019.02666

**Published:** 2019-11-20

**Authors:** Clarissa Schwab, Evelyn Voney, Alejandro Ramirez Garcia, Michaela Vischer, Christophe Lacroix

**Affiliations:** ^1^Laboratory of Food Biotechnology, Department of Health Sciences and Technology, ETH Zürich, Zurich, Switzerland; ^2^Medela AG, Baar, Switzerland

**Keywords:** human breast milk, strict anaerobes, cultivation, storage, metabolites

## Abstract

Besides nutritional components, breast milk contains diverse microbes, which may be involved in colonization of the infant gut. Expressed milk is often stored for few days in the refrigerator. The aim of this study was to determine the abundance, prevalence and diversity of facultative and strict anaerobic bacteria using culture-dependent and -independent methods, and to determine changes in milk microbial and chemical composition during storage. Samples of mature breast milk from 21 women were collected 3–6 months post-partum and were analyzed fresh or after anaerobic storage for 6 days at 4°C. The cultivable bacterial population was analyzed using the most probable number (MPN) method or plate counts and different media. The abundance of major bacterial groups was determined using quantitative PCR and 16S rRNA gene sequencing. Lactose, lactate, short chain fatty acids (SCFA) and human milk oligosaccharides (HMO) were analyzed using chromatography techniques. Highest mean viable cell counts were obtained in yeast casitone fatty acids (YCFA) broth supplied with mucin (log 4.2 ± 1.8 cells/ml) and lactose (log 3.9 ± 1.4 cells/ml), or Columbia broth (log 3.0 ± 0.7 cells/ml). Mean total bacterial counts estimated by qPCR was 5.3 ± 0.6 log cells/ml, with *Firmicutes* being the most abundant phylum. The most prevalent bacterial groups were *Streptococcus* spp. (15/19 samples), *Enterobacteriaceae* (13/19) and *Lactobacillus/Lactococcus/Pediococcus* group (12/19). While the average total number of bacterial cells did not change significantly during storage, the prevalence of strict anaerobic *Bacteroidetes* increased threefold, from 3/19 to 9/19, while in 7 samples *Clostridium* clusters IV or XIVa became detectable after storage. Major HMO were not degraded. Lactate was present in 18/21 samples after storage (2.3–18.0 mM). Butyrate was detected in 15/21 and 18/21 samples before and after storage, respectively, at concentrations ranging from 2.5 to 5.7 mM. We demonstrate enhanced prevalence and/or abundance of viable strict anaerobes from the *Bacteroidetes* and *Clostridiales* after 6-day anaerobic storage of human milk. Our data indicate that anaerobic cold storage did not markedly change total viable bacterial load, while HMO profiles were stable. Anaerobic cold storage of human milk for up to 6 days may be suitable for preserving milk quality for potential microbial transfer to the infant gut.

## Introduction

Breast milk harbors microbes that are potentially transferred to the neonate (Heikkila and Saris, 2003; Jeurink et al., 2013) and may influence the initial neonatal gut colonization and the maturation of the immune system (Fernández et al., 2013). The composition of the human milk microbiota was shown to depend on mode of delivery, lactation time, gestational age and also geographical locations (Khodayar-Pardo et al., 2014; Cabrera-Rubio et al., 2016; Kumar et al., 2016), the latter of which has not been confirmed by all studies (Li et al., 2017).

Studies using cultivation-dependent techniques mainly isolated facultative anaerobes of the genera *Staphylococcus, Streptococcus, Lactococcus, Leuconostoc, Weissella, Enterococcus, Lactobacillus*, *Cutibacterium* (formerly *Propionibacterium*) and *Enterobacteriaceae* (Jeurink et al., 2013; Jost et al., 2013). The oxygen tolerance of bifidobacteria, which have also been isolated, varies between species (Andriantsoanirina et al., 2013). Accordingly, molecular techniques based on amplification of the 16S rRNA gene (quantitative PCR or 16S rRNA gene amplicon sequencing) indicated a high abundance of *Firmicutes* and *Proteobacteria*, and frequently recovered *Actinobacteria* (Urbaniak et al., 2016; Moossavi et al., 2019). In addition, 16S rRNA gene amplicon sequencing suggested the presence of strict anaerobes of the orders *Clostridiales* and *Bacteroidales* in breast milk, albeit at low abundance (Collado et al., 2009; Jost et al., 2013; Murphy et al., 2017; Moossavi et al., 2019). The presence of viable strict anaerobic gut microbes has, however, not been confirmed by cultivation (Jost et al., 2013). Several pathways have been suggested that explain the bacterial transfer to breast milk. Bacteria can enter milk from the skin of the mother or from the oral cavity of the infant. Another hypothesis is an active migration of gut bacteria to the mammary gland through an endogenous route (Jeurink et al., 2013). The isolation of identical strains of *Bifidobacterium* spp. in mother-infant pairs (Jost et al., 2014; Murphy et al., 2017) supported this second pathway.

The World Health Organization recommends to exclusively breastfeed newborns up to 6 months post-partum (World Health Organization, 2001, 2003). Mature milk of term infants generally contains 67–78 g/L lactose, 32–36 g/L fat, and 9–12 g/L proteins. This composition seems to be conserved across populations (Ballard and Morrow, 2013) but concentrations vary between mothers and over lactational stages (Fleischer Michaelsen et al., 1990), diurnally and within one feeding (Gunther et al., 1949; Hall, 1979), and outliers have been reported (Ballard and Morrow, 2013). The main carbohydrate in milk is lactose, HMO are the second most abundant carbohydrate source with concentrations of 10–20 g/L (Petherick, 2010). HMO are composed of monosaccharides D-glucose, D-galactose, L-fucose, *N*-acetyl-D-glucosamine, and *N*-acetylneuraminic acid (also sialic acid). The most abundant secreted HMO include lacto-N-tetraose, lacto-*N*-neotetraose (LNnT), 2′fucosyllactose (2′FL), 3′fucosyllactose (3′FL), and the acidic HMO 3′sialyllactose (3′SL) and 6′sialyllactose (6′SL) (Kunz et al., 2000; Garrido et al., 2015). Breast milk provides essential amino and fatty acids, and long-chain polyunsaturated fatty acids. Lipids present in human milk cover 40–50% of the energy needs of the suckling infant (Yuhas et al., 2006). Beside these macronutrients, bioactive molecules such as secretory antibodies, immune cells, antimicrobial proteins like lactoferrin and lysozymes, and regulatory cytokines are part of human milk (Newburg, 2005).

Breast milk is suckled by the infant, or is expressed by mechanic or electric breast pumps and frequently stored aerobically at 4 or −20°C before feeding (Rasmussen and Geraghty, 2011), which may lead to degradation of some nutrients and milk microbes (Marín et al., 2009; Cabrera-Rubio et al., 2012). These storage conditions would not allow the preservation of strict anaerobic bacteria. It was therefore the aim of this study to investigate microbiological stability during anaerobic milk storage with a focus on the cultivable microbiota. We tested the metabolism of the major sugars, lactose and HMO, as substrate carbohydrates for bacterial fermentation in breast milk and for the gut microbiota, resulting in the production of SCFA.

## Materials and Methods

### Subjects and Sampling Conditions

A total of 21 breastfeeding women (27–40 years old) with infants aged 2.3–5.5 months were recruited in Switzerland. Exclusion criteria included the intake of antibiotics or probiotics within 1 month before the sampling day, chronic and acute diseases like fever, inflammation, diarrhea, and mastitis. Breast milk samples were collected from one breast lobe at one time point especially for this study. Written informed consent was obtained from the participants. The study protocol was approved by the Ethics Committee of the ETH Zurich (2018-N-14).

Prior collection, nipples and surroundings of one breast were cleaned using HibiScrub (Swissmedic, Switzerland) to reduce bacteria load on the skin. Milk samples (approximately 10–15 mL) were collected in a sterile flask wearing gloves and using an electrical pump (Medela, Switzerland). The first 3–5 ml were discarded. After expression, samples were immediately placed in an anaerobic jar (Oxoid AG, Switzerland) containing the Anaerocult A system (Merck, Switzerland) and transported to an anaerobic chamber (10% CO_2_, 5% H_2_, 85% N_2_, Coy Laboratories, United States). Samples were aliquoted for the different analyses or storage assays, half of the aliquots were used for immediate cultivation or were centrifuged at 14,000 × *g* for 20 min at 4°C. Cell pellets and supernatants were frozen at −20°C until further analysis. Other aliquots were transferred to an anaerobic jar containing the Anaerocult A system (Merck), were stored at 4°C for 6 days and then processed as described above (stored samples). The pH of fresh and stored milk samples was determined using a pH meter (Mettler-Toledo AG, Greifensee, Switzerland).

### Cultivation Medium

Columbia Broth (Becton Dickinson, Switzerland), which is a general medium used for the cultivation of fastidious bacteria was produced aerobically (Col_ae) according to the instructions of the supplier, or anaerobically (Col_an) to cultivate facultative anaerobes and strict anaerobes. Therefore, the Columbia powder was mixed with distilled water, boiled for 15 min, cooled down and flushed with CO_2_. L-cysteine (Sigma-Aldrich, Switzerland) was added when the temperature of the medium was approximately 50°C. Columbia broth was transferred to Hungate tubes flushed with CO_2_, tubes were sealed, and autoclaved. Yeast extract, casitone and fatty acid (YCFA) medium is used to cultivate gut microbes that depend on the presence of volatile fatty acids for growth (Duncan et al., 2002; Supplementary Table S1). We adapted the composition of the fatty acid mix to acetate (70%), lactate (25%) and propionate (5%) to mimick the metabolite profile present in the infant gut (Pham et al., 2016). YCFA was prepared without added carbohydrate (YCFAwo) or with 18 g/l lactose (YCFA + Lact) or 2 g/l porcine mucin (YCFA + Muc). All components except L-cysteine-HCl were solubilized in deionized water, and the pH was adjusted to 7.6 with NaOH. The medium was flushed with CO_2_ and boiled. When the color changed from blue to pink, L-cysteine-HCl was added. The medium (10 ml) was transferred to Hungate tubes flushed with CO_2_, and tubes were sealed and autoclaved. Reinforced clostridial broth (RC; Sigma-Aldrich) and *Bacteroides* Mineral Salt Broth (BMS; Jost et al., 2013) were prepared in Hungate tubes according to instructions of the suppliers. Cultivation of lactic acid bacteria was carried out on agar plates with using modified de Man-Rogosa-Sharpe (mMRS) medium (Stolz et al., 1995), with 1.5% agar (Difco). Modified Wilkins-Chalgren (mWC) agar plates containing 100 mg/l norfloxacin and 100 mg/l mupirocin were used to isolate *Bifidobacterium* spp. (Vlková et al., 2015).

### Cultivation Conditions for Viable Cells

Cultivable cells were assessed in freshly expressed and stored breast milk using the most probable number (MPN) technique. A ten-replicate design (Sutton, 2010) adapted to 96 well microtiter plates (Kuai et al., 2001) was used. Milk samples were diluted with anaerobically prepared phosphate buffered saline (8 g/l NaCL, 200 mg/l KCl, 1.44 g/l Na_2_HPO_4_, 240 mg/l KH_2_PO_4_; pH 7.4) in tenfold dilution series. For each dilution, 180 μl of medium were supplied in microtiter plate (Bioswisstec AG, Switzerland) to which 20 μl of diluted sample were added. Microtiter plates were covered with a lid, and incubated at 37°C for 48 h inside the anaerobic chamber. For determination of MPN of aerobically grown cells, microtiter plates were prepared outside the anaerobic chamber and were incubated aerobically at 37°C for 48 h. Before and after incubation the optical density at 590 nm (OD_590_) was determined (Powerwave XS, BioTek). An increase in OD_590_ of ≥0.1 compared to the corresponding baseline was considered growth, and was confirmed visually. Based on these OD_590_ data, the MPN was calculated using the Excel Spread Sheet provided by the Food and Drug Administration (FDA, BAM^1^). Following the OD_590_ measurement, 5 replicates of the 10^–1^ dilution were pooled and centrifuged at 13,000 × *g* for 15 min. Supernatants and pellets were kept at −20°C until further analysis. To determine the cultivable lactic acid bacteria and bifidobacteria, 100 or 200 μl of fresh or stored milk and ten-fold dilutions were spread on modified mMRS or mWC agar plates inside the anaerobic chamber in duplicates, and were incubated anaerobically for 2–4 days at 37°C.

To identify colonies from agar plates, DNA was isolated from liquid culture as described below, and the 16S rRNA gene was amplified using PCR and primers F8 (5′-AGA GTT TGA TCM TGG CTC-3′), and 1391R (5′-GAC GGG CGG TGT GTR CA-3′), both 10 μM, Microsynth, Switzerland). Amplicons were sent for Sanger sequencing to Eurofins Genomics (Germany). The resulting sequences were aligned against the 16S rRNA gene database provided by NCBI using blastN.

### DNA Isolation

Cell pellets obtained from fresh and stored breast milk samples and from bacterial cultures (1 ml) were thawed and the DNA extraction was done using Fast DNA SpinKit for Soil which includes a bead beating step (MP Biomedicals, France). DNA concentration was determined using QuBit and the dsDNA HS Assay Kit (Thermo Fisher), and ranged from 90 pg/ul to 39.3 ng/μl, whereas three samples were below detection limit (<0.5 pg/μl) (Supplementary Table S2). DNA samples were stored at −20°C until further analysis.

### Quantitative PCR (qPCR)

Quantification of total bacteria and selected bacterial groups in milk samples before and after cold storage was performed using a Roche Light Cycler 480 (Table 1). Reactions contained 5 μl 2× SensiFASTSYBR (Labgene Scientific Instruments, Switzerland), 0.5 μl forward and reverse primer (each 10 μM; Microsynth), 3 μl MilliQ water, and 1 μl DNA. During the thermal cycling, the reaction mixtures were initially heated at 95°C for 3 min, followed by 40 cycles of denaturation at 95°C for 5 s, and annealing and extension at 60°C for 30 s. Melting curve analysis using a melting interval from 65 to 95°C was conducted to verify amplification specificity. All samples were analyzed in duplicates and in each run a standard curve out of a 10-fold dilution series of a linearized plasmid harboring the gene of interest was included. To correct for multiple 16S rRNA gene copies in one bacterial cell, gene copies were corrected for total bacteria, *Firmicutes*, *Bacteroidetes, Lactobacillus/Leuconostoc/Pediococcus* spp. (all correction factor 5) and *Veillonella* spp., *Clostridium* cluster IV (both correction factor 4) and XIVa (correction factor 6) (Stoddard et al., 2015).

**TABLE 1:** Targets and primers used in qPCR reactions.

**Bacterial phylum**	**Target genes**	**Primer**	**5**′**-3**′	**References**
-	Total bacteria 16S rRNA gene	Eub338F	ACWCCTACGGGWGGCAGCAG	Guo et al., 2008
		Eub518R	ATTACCGCGGCTGCTGG	
*Firmicutes*	*Firmicutes* 16S rRNA gene	Firm934F	GGAGYATGTGGTTTAATTCGAAGCA	Guo et al., 2008
		Firm1060R	AGCTGACGACAACCATGCAC	
	*Clostridium* cluster IV 16S rRNA gene	Clep866mF	TTAACACAATAAGTWATCCACCTGG	
		Clept1240mR	ACCTTCCTCCGTTTTGTCAAC	Ramirez-Farias et al., 2009
	*Clostridium* cluster XIVa 16S rRNA gene	g.Ccoc-F g-Ccoc-R	AAATGACGGTACCTGACTAA CTTTGAGTTTCATTCTTGCGAA	Matsuki et al., 2004
	*Veillonella* spp. 16S rRNA gene	Vspp - F	AYCAACCTGCCCTTCAGA	Price et al., 2007
		Vspp - R	CGTCCCGATTAACAGAGCTT	
	*Lactobacillus/Pediococcus/Leuconostoc* 16S rRNA gene	F_Lacto 05	AGCAGTAGGGAATCTTCCA	Furet et al., 2009
		R_Lacto 04	CGCCACTGGTGTTCYTCCATATA	
	*Streptococcus* spp. *tuf*	Tuf-Strep-1	GAAGAATTGCTTGAATTGGTTGAA	Collado et al., 2009
		Tuf-Strep-R	GGACGGTAGTTGTTGAAGAATGG	
	*Staphylococcus* spp. *tuf*	T-Stag422	GGCCGTGTTGAACGTGGTCAAATCA	Martineau et al., 2001
		TStag765	TYACCATTTCAGTACCTCTGGTAA	
*Bacteroidetes*	*Bacteroides/Prevotella/Porphyromonas* group 16S rRNA gene	Bac303F	GAAGTCCCCCACATTG	Ramirez-Farias et al., 2009
		Bfr-Femrev	CGCKACTTGGCTGGTTCAG	
*Proteobacteria*	*Enterobacteriaceae* 16S rRNA gene	Eco1457F	CATTGACGTTACCCGCAGAAGAAGC	Bartosch et al., 2004
		Eco1652R	CTCTACGAGACTCAAGCTTGC	
*Actinobacteria*	*Bifidobacteria* 16S rRNA gene	Bif F	TCGCGT CYGGTGTGAAAG	Rinttilä et al., 2004
		Bif R	CCACATCCAGCRTCCAC	
	*Propionibacterium*/*Cutibacterium* spp. 16S rRNA gene	Avi Fwd	GTCTGCAACTCGACCCCAT	Rocha Martin et al., 2018
		Avi Probe	CTTCGACGGCTCCCCCACACAGGT	

### 16S rRNA Gene Library Preparation and Sequencing

Libraries covering the V3 region of the 16S rRNA gene were prepared using a two-step PCR approach. In the first reaction, 100 ng DNA was used as template and was amplified with 12 μl AccuPrime SupermixII (Life Technologies Europe BV, Switzerland), 0.5 μl of with barcoded primers NXt_338F (5′-TCG TCG GCA GCG TCA GAT GTG TAT AAG AGA CAG ACW CCT ACG GGW GGC AGC AG-3′) and NXt_518R (5-′GTC TCG TGG GCT CGG AGA TGT GTA TAA GAG ACA GAT TAC CGC GGC TGC TGG-3′; Microsynth, both 10 μM) and water to a final volume of 25 μl. Thermocycling was conducted as follows: initial denaturation at 95°C for 2 min, 25 cycles of denaturation at 95°C for 15 s, annealing at 55°C for 15 s and extension at 68°C for 30 s followed by final extension at 68°C for 4 min.

To add adapters and indexes, a second PCR was performed using 12 μl of AccuPrime Pfx Fusion mix (Thermo Fisher), 2 μl of corresponding P5 and P7 primer (Nextera Index Kit, Illumina, United States), 2 μl of the PCR product from the first PCR and nuclease-free water to a final volume of 25 μl. The PCR temperature profile was as follows: 98°C for 1 min, followed by 12 cycles of: 95°C for 15 s, 55°C for 20s and 72°C for 20 s and a final extension step at 72°C for 5 min. The amplified fragments were purified using AMPure XP beads (Beckman Coulter Genomic, United States) according to manufacturer’s manual. Prior to pooling, libraries were quantified using a Qubit fluorometer (Thermo Fisher) and were mixed in approximately equal concentrations.

Due to low concentration of DNA in most milk samples (Supplementary Table S2), low efficiency of the barcoded degenerated primers used for amplicon sequencing, and the likely presence of contaminating eukaryotic DNA, the standard sequencing protocol had to be adapted to a nested PCR approach, similar as previously employed for breast milk or other samples from environments with low bacterial load (Yu et al., 2015; Boix-Amorós et al., 2016; Kostric et al., 2018). For the optimized protocol, the V3 hypervariable 16S rRNA gene region of selected milk samples was preamplified with primers 338F (10 μM) and 518R (10 μM) as described above, using 5 μl of milk DNA as template. Purified PCR products (WizardSV Gel and PCR Clean-Up System, Promega, Switzerland) were then used for the barcoding PCR.

Sequencing was carried out on an Illumina MiSeq at the Genetic Diversity Center of ETH Zurich. Preparation of the library used the V2 2 × 250 bp paired end NextTera chemistry supplemented with 30% of PhiX. Raw sequencing reads were processed by merging the paired reads using USEARCH (v8.1.1756) with to a minimum length of 100 bp setting the expected error threshold to 1 and the minimum overlapping to 15 bp. Raw sequencing reads were filtered using PRINSEQ-lite (v0.20.4). Sequences that passed quality filtering were clustered into OUTs at 97% identity level using UPARSE (usearch v8.0.1623). Representative sequences were aligned using PyNAST (QIIME-1.8.0) and taxonomically assigned using UTAX (usearch v8.1.1756). Sequencing of MPN samples (*n* = 12) yielded 584,096 reads (mean 48,675 reads/sample), sequencing of milk samples (*n* = 6) yielded 148,590 reads (mean 24,765 reads/sample).

### Lactose, Lactate and SCFA Determination

The supernatants from fresh and stored milk, and from randomly selected MPN milk samples were subjected to high performance liquid chromatography (Merck-Hitachi, Switzerland) with refractive index detection (HPLC-RI) using an Aminex HPX-87H column (300 × 7.8 mm; BioRad, Switzerland). Thawed supernatants (40 μl) were, without additional extraction procedure, eluted at 40°C with 10 mM H_2_SO_4_ at a flow rate of 0.6 ml per minute. Lactose, SCFA and lactate were quantified using external standard (all from Sigma-Aldrich). Detection limits were 1 mM for lactose, 0.9 mM for lactate, and 0.5 mM for formate, succinate, acetate, propionate and butyrate. A representative chromatogram is shown in Supplementary Figure S1.

### Qualitative Analysis of HMO Composition of Fresh and Stored Milk

HMO profiles before and after milk storage were qualitatively analyzed using high pH anion exchange chromatography with amperometric detection (HPAEC-PAD) on a Dionex IC3000 equipped with a CarbopacPA20 column (Thermo Fisher) and an electrochemical detector with a gold electrode. HMO were analysed directly from milk supernatants without further extraction procedure. Water (A), 200 mM NaOH (B) and 1 M Na-acetate (C) were used as solvents at a flow rate of 0.25 ml/min with the following gradient: 0 min 30.4% B, 1.3% C, 22 min 30.4% B, 11.34% C, followed by washing and regeneration. Main HMO were identified using 2′FL, 3′FL, 3′SL, 6′SL, lacto-*N*-neotetraose, *N*-acetylneuraminic acid supplied by Glycom A/S (Denmark) as external standards.

### Statistical Analysis

Differences in log viable counts between fresh and stored samples, and between cultivation media were tested using paired two-tailed *t*-test or Kruskal Wallis One Way Analysis of Variance of Ranks with Dunn’s method for pairwise comparison (SigmaPlot 13), respectively. Differences between cell counts in fresh and stored samples determined using qPCR were evaluated using the paired two-tailed *t*-test. The degree of correlations between parameters and their significance was tested using IBM SPSS Statistics 20.0 (IBM SPSS Inc., Chicago, IL, USA). Significance was set at *p* < 0.05.

### Data Availability

16S rRNA gene sequences are available at ncbi’s sra archive under bioproject accession number PRJNA557136.

## Results

### Cultivable Bacteria in Fresh and Stored Human Breast Milk Samples

**FIGURE 1:**
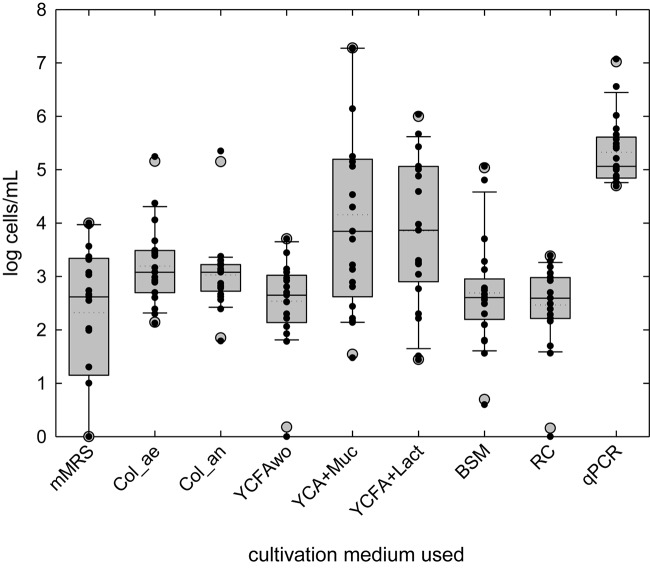
Bacterial load in fresh breast milk investigated by cultivation or quantitative PCR. Boxes indicate the 25th and 75th percentiles. Whiskers indicate 10th and 90th percentiles, 5th and 95th percentiles are shown as dots. The dotted and solid lines indicate mean and median, respectively. (Viable) cell counts of individual samples are indicated by black dots.

The viable cell counts in fresh and cold stored breast milk were assessed using a cultivation-based approach combining MPN technique and cultivation on agar plates with different semi- and non-selective media. Significant higher mean cell counts in fresh breast milk were obtained in YCFA containing either mucin or lactose (YCFA + Muc: log 4.2 ± 1.8 cells/ml, YCFA + Lact: log 3.9 ± 1.4 cells/ml), or aerobic (log 3.2 ± 0.7 cells/ml) and anaerobic (log 3.0 ± 0.7 cells/ml) Columbia medium compared to the other media tested (Table 2 and Figure 1). The addition of lactose and mucin led to significantly higher cell counts compared to YCFAwo (log 2.5 ± 0.8 cells/ml). Lactic acid bacteria viable counts on mMRS agar plates were log 2.3 ± 1.3 CFU/ml in fresh samples (Figure 1). Representative isolates obtained from the undiluted samples were identified as *Enterococcus* and *Streptococcus* spp., and *Cutibacterium acnes*. No growth was observed on mWC agar plates used to detect bifidobacteria.

**TABLE 2:** Mean viable cell counts in fresh and anaerobic milk samples.

**Medium**	**mMRS**	**mWC**	**Columbia aerobic**	**Columbia anaerobic**	**YCFAwo**	**YCFA + Muc**	**YCFA + Lac**	**BMS**	**RC**
							
**Sample**	**(log CFU/ml)**				**(log cells/ml)**				
Fresh	2.3 ± 1.3^b,A^	nd^1^	3.2 ± 0.7^a,A^	3.0 ± 0.7^a,A^	2.5 ± 0.8^b,A^	4.2 ± 1.8^a,A^	3.9 ± 1.4^a,A^	2.7 ± 1.0^b,A^	2.5 ± 0.8^b,A^
Stored	2.1 ± 1.4^b,A^	nd	2.9 ± 1.0^b,A^	3.0 ± 0.8^b,A^	3.2 ± 1.0^*b, B*^	4.6 ± 1.4^a,A^	4.3 ± 1.5^a,A^	3.3 ± 1.9^a,A^	2.4 ± 1.0^b,A^

*Mean viable cell counts in fresh and anaerobically stored (6 days at 4°C) breast milk samples (*n* = 21) were determined by MPN or plate counts using different cultivation media. ^1^nd, not detected. ^*a,b*^Mean viable cell counts of different cultivation media fresh or stored samples sharing the same small letter did not significantly differ. ^*A,B*^Mean viable cell counts fresh and stored samples determined in one media type sharing the same capital letter did not significantly differ.*

During storage, mean cell counts significantly increased in YCFAwo (*p* < 0.05, Δlog 0.6 ± 0.8 cells/ml) compared to fresh milk samples, but no significant change was measured for the other cultivation conditions (Supplementary Figure S2 and Table 2). No significant correlations were detected for viable cell concentrations among the different culture conditions, suggesting that each cultivation medium supported different groups of bacteria, with the exception of cell counts in YCFA + Muc and YCFA + Lact which positively correlated (*r* = 0.385; *p* < 0.05).

After grouping data for infants according to age (≤3 months, average 2.8 ± 0.4 months, *n* = 9; and >3 months, average 4.8 ± 0.6 months, *n* = 12), log MPN were significantly higher for stored samples tested in anaerobic Columbia Broth (≤3 months: log 2.6 ± 0.7 cells/ml, >3 months: log 3.2 ± 0.6 log/ml; *p* < 0.05) and in YCFA + Lact (≤3 months: log 5.1 ± 1.4 log cells/ml, >3 months: 4.0 ± 1.2 log cells/ml; *p* < 0.05). No group difference was detected for other media and for fresh samples in all media. Age of the infants negatively correlated with fresh milk bacterial cell counts in RC (*r*: −0.54, *p* < 0.05), and mMRS (*r*: −0.46, *p* = 0.037) and YCFA + Lact (*r*: −0.47, *p* < 0.05), and positively with bacterial load in Col_an (*r*: 0.64 *p* = 0.034) of stored samples. The age of the mother was negatively correlated with log MPN after cold storage in RC medium (*r*: −0.54; *p* = 0.013).

### Bacterial Composition and Production of SCFA and Lactate During the Cultivation in Different Media

Supernatants of selected samples grown in YCFAwo, and YCFA + Muc were analyzed by HPLC-RI to investigate the formation of SCFA. In YCFAwo (*n* = 6 samples analyzed), formation of acetate (42.4 ± 8.7 mM), lactate (11.9 ± 1.4 mM) and propionate (2.3 ± 0.4) was detected in 5/6 samples. In YCFA + Muc in 6/12 samples from day 0, lactate (7.9–68.2 mM), acetate (3.4–13.2 mM) and formate (3.1–7.3 mM) were formed, propionate was detected in one sample (12.1 mM). In two samples, this metabolic profile differed at day 6 with lactate utilization (−5.7 to −6.9 mM) observed while the other three samples showed reduced lactate and/or acetate formation. In four fresh milk samples, lactate was used (−6.0 to −11.2 mM) and acetate was formed (6.2–19.3 mM) with little difference between day 0 and day 6. For one sample, metabolic activity (lactate formation and acetate utilization) was only observed at day 0, while for one sample there was little metabolic activity at both sampling days.

**FIGURE 2:**
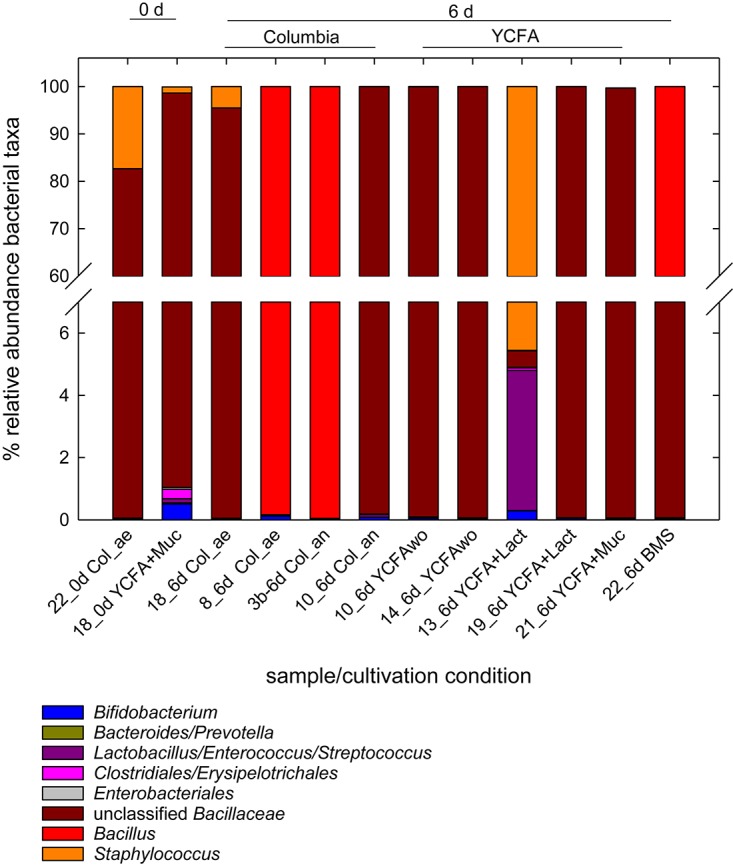
Relative abundance of bacterial taxa in selected MPN samples. Samples were collected from 10 fold dilutions of fresh and stored milk samples grown for 24 h at 37°C in MPN assays conducted in different media. The MPN of these samples ranged from log 2.8 cells/mL to log 6.3 cells/mL). Microbiota composition was determined using 16S rRNA gene amplicon sequencing.

We also determined the 16S rRNA gene composition of selected MPN samples in overnight cultures of the −1 dilutions with total cell counts ranging from log 2.8–7.3 cells/mL (Figure 2). Unclassified *Bacillaceae*, *Bacillus*, and *Staphylococcus* were the most abundant taxa contributing >95% of the reads. *Bifidobacterium* were present in all analyzed samples at <0.6% relative abundance. Strict anaerobes of the *Clostridiales* and the *Bacteroidales* were recovered at around 0.35 and 0.01%, respectively. The alpha-diversity within each sample was low as estimated using the number of observed OTUs, Shannon diversity and Chao1 index as measures. The number of observed OTUs (12–23) correlated positively with MPN of the sample (*p* < 0.05). The Shannon index, which equally weighs richness and evenness, ranged from 0.01 to 0.67, while Chao1 indices ranged from 10 to 42.

### Abundance and Diversity of Bacterial Population of Fresh and Stored Breast Milk

**FIGURE 3:**
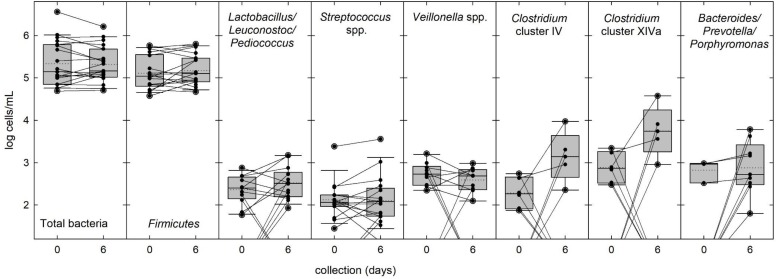
Bacterial populations in fresh and stored breast milk samples. The abundance of total bacteria and of selected bacterial groups commonly present in breast milk was determined by qPCR. Boxes indicate the 25th and 75th percentiles. Whiskers indicate 10th and 90th percentiles, 5th and 95th percentiles are shown as dots. The dotted and solid lines indicate mean and median, respectively. Cell counts of individual samples and changes in abundance during storage are indicated by black dots and connecting lines.

Breast milk samples (*n* = 19) were analyzed by qPCR to estimate cell counts of total bacteria and of selected bacterial groups representing the predominant phyla *Firmicutes*, *Bacteroidetes*, *Actinobacteria*, and *Proteobacteria* in fresh and stored milk, using correction for multiple 16S rRNA gene copies per cell. Fresh and stored milk samples contained an average of total bacteria of log 5.4 ± 0.5 cells/ml (Figure 3 and Supplementary Table S3). Only limited changes in total bacteria were observed with a maximum two-fold increase of samples 8, 9, and 10 (Δlog 0.3 cells/ml breast milk), while highest overall decrease was detected in samples 5, 18, 20, and 21 (Δlog −0.4 cells/ml breast milk) (Figure 3 and Supplementary Table S3).

In fresh milk, the most prevalent phylum was *Firmicutes* contributing the majority of cells (85%) in all samples. *Streptococcus* spp*., Lactobacillus/Leuconostoc/Pediococcus* spp. and *Veillonella* spp. were present in 79, 63, and 53% of the fresh samples, respectively (Figure 3 and Supplementary Table S3), while *Staphylococcus* spp., *Clostridium* clusters IV and XIVa were only detected in 26% of the samples. Mean cell counts of these major bacterial groups did not significantly change during storage (Figure 3 and Supplementary Table S3). *Enterobacteriaceae* (phylum *Proteobacteria*) were detected in 68 and 58% of the samples before and after storage, respectively, at low abundance. *Propionibacterium/Cutibacterium* spp. was detected in 5 and 16% of the samples before and after storage, respectively, and *Bifidobacterium* 16S rRNA genes (both phylum *Actinobacteria*) could not be amplified in any sample. After storage, the prevalence of *Bacteroides/Prevotella/Porphyromonas* group was threefold increased from 16 to 47% (Figure 3 and Supplementary Table S3). There was no change in prevalence and abundance of *Clostridium* cluster XIVa while the abundance of *Clostridium* cluster IV significantly increased (Supplementary Table S3).

To investigate bacterial diversity, we tested 16S rRNA gene amplicon sequencing. As the standard protocol did not yield and PCR products, we employed a nested PCR approach. Visible bands after the initial and the 1st PCR were obtained for samples that contained >2 ng/μL DNA, or >log 6 cells/ml, which corresponded to only six samples in total. However, this approach yielded a high proportion (13–70%) of unclassified reads even at phylum level (Supplementary Figure S3) indicating unspecific overamplification. The most abundant assigned phyla were *Firmicutes* (*Streptococcaceae, Staphylococcaceae, Peptostreptococcaceae*, and *Erysipelotrichaceae*) and *Proteobacteria* (*Burkholderiaceae, Comamonadaceae*, and *Oxalabacteraceae*), and at lower relative abundance *Actinobacteria* (*Bifidobacteriaceae*).

### Impact of Storage on Lactose, Human Milk Oligosaccharide and SCFA Profile of Breast Milk

Breast milk samples were analyzed by HPLC-RI to determine the concentrations of lactose, SCFA and lactate. The average lactose content in fresh milk samples was not impacted by milk storage, with 161.8–235.8 mM and 156.3–227.6 mM in fresh and stored milk, respectively (Table 3). Lactose content decreased in 9 stored milk samples (−43.8 to −5.2 mM) but increased in 11 samples (+ 1.8–15.8 mM), respectively (Table 3). Lactate was detected after anaerobic cold storage in 18/21 samples at concentrations, ranging from 2.3 to 18 mM, but was not present in fresh milks. Lactate concentration in stored milks negatively correlated with the change of pH during storage (from −1.4 to 0 pH unit); *r*: −0.529, *p* = 0.016) but was not correlated with the lactose concentration change. Free butyrate was detected in 15/21 and 18/21 samples before and after storage, respectively, at concentrations in the range from 2.5 to 5.7 mM. Acetate, propionate or formate were not present in detectable amounts in any sample.

**TABLE 3:** Lactose, lactate, and butyrate concentration and pH in fresh and stored breast milk samples.

**ID**	**Sugar or metabolite concentration (mM)**									**pH**		
		
	**Lactose**			**Lactate**			**Butyrate**					
						
	**Fresh**	**Stored**	**Δ**	**Fresh**	**Stored**	**Δ**	**Fresh**	**Stored**	**Δ**	**Fresh**	**Stored**	**Δ**
2	235.8	na	nd	nd	3.1	+ 3.1	nd	nd	–	7.0	6.4	−0.6
3	214.7	209.5	−5.2	nd	18.0	+ 18.0	nd	5.7	+ 5.7	na	na	na
4	257.1	213.3	−43.8	nd	7.0	+ 7.0	5.3	5.1	−0.2	6.8	6.3	−0.5
5	227.6	212.7	−14.9	nd	2.7	+ 2.7	nd	5.7	+ 5.7	6.7	6.4	−0.3
6	201.3	206.5	+ 5.2	nd	nd	–	5.7	5.5	−0.2	6.9	6.7	−0.2
7	231.1	260.0	+ 28.9	nd	3.6	+ 3.6	4.8	4.6	−0.2	6.8	6.7	−0.1
8	200.7	206.8	+ 6.1	nd	5.8	+ 5.8	4.6	3.9	−0.7	6.7	6.5	−0.2
9	205.4	217.4	+ 12.0	nd	7.6	+ 7.6	4.4	4.6	−0.2	7.1	6.5	−0.6
10	222.3	213.6	−8.8	nd	5.6	+ 5.6	4.1	3.9	−0.2	7.3	6.6	−0.7
11	223.2	224.9	+ 1.8	nd	8.1	+ 8.1	4.6	3.7	−0.9	6.7	6.3	−0.4
12	207.7	216.2	+ 8.5	nd	6.7	+ 6.7	3.9	4.4	+ 0.5	7.7	6.3	−1.4
13	231.7	231.7	0	nd	nd	nd	3.9	4.1	+ 0.2	7.0	7.0	0
14	209.5	220.6	+ 11.1	nd	5.4	+ 5.4	3.7	3.2	−0.5	7.6	6.4	−1.2
15	204.5	192.8	−11.7	nd	5.6	+ 5.6	3.4	3.4	0	7.2	6.3	−0.9
16	198.9	191.9	−7.0	nd	7.4	+ 7.4	3.2	3.0	−0.2	7.3	6.4	−0.9
17	217.6	220.0	+ 2.3	nd	2.3	+ 2.3	2.8	3.2	+ 0.4	7.2	6.6	−0.6
18	176.5	192.2	+ 15.8	nd	6.3	+ 6.3	3.0	2.5	−0.5	7.2	6.1	−1.1
19	208.6	214.1	+ 5.6	nd	5.2	+ 5.2	nd	2.5	+ 2.5	7.1	6.3	−0.8
20	212.1	223.5	+ 11.4	nd	2.3	+ 2.3	2.5	nd	−2.5	6.7	6.8	+ 0.1
21	213.6	229.3	+ 15.8	nd	6.1	+ 6.1	nd	nd	–	7.3	6.5	−0.8
22	161.8	156.3	−5.6	nd	nd	nd	nd	2.8	2.8	7.4	7.0	−0.4

*Lactose, lactate and butyrate concentration in fresh and stored samples from 21 mothers was measured by HPLC-RI. nd: not detectable; na: not available*

**FIGURE 4:**
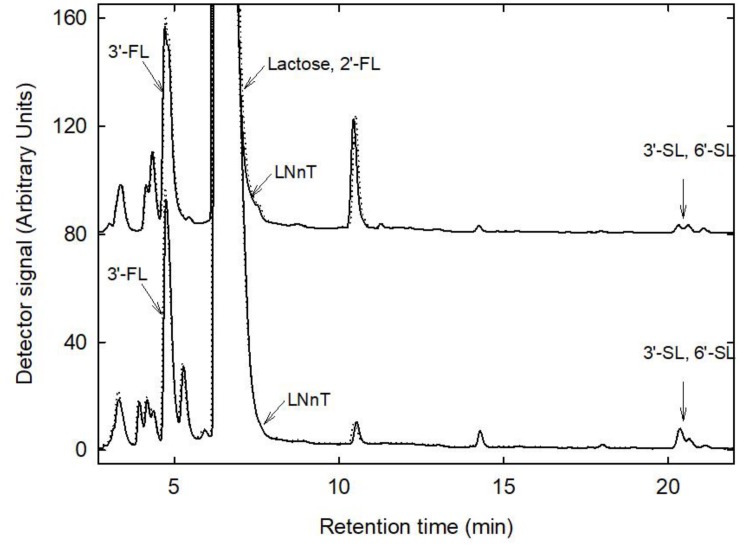
HMO profiles before and after storage. Shown are representative profiles generated from fresh (bold line) and stored (dotted line) samples 12 and 13. Major HMO were identified using external standards.

HMO profiles were generated from fresh and stored breast milk samples using HPAEC-PAD (Figure 4). The major HMOs 3′FL, 3′SL, 6′SL were detected in all samples. 2′FL could not be analyzed due to similar retention time as lactose, LNnT appeared as a shoulder of the lactose peak. There was no change in HMO profiles, no visible release of fucose or sialic acid (*N*-acetylneuraminic acid), and no accumulation of glucose or galactose, indicating that at least the most prevalent and abundant HMO were not degraded during storage.

## Discussion

Human breast milk banks usually store samples aerobically at −20°C to minimize or prevent biological and chemical degradation, after batchwise pasteurization is performed (Haiden and Ziegler, 2016). These treatments likely inactivate a large fraction of the non-spore forming microbes present in fresh milk. Indeed, previous studies observed different impact of freezing on microbiota composition (Martin et al., 2009; Ahrabi et al., 2016). In this study, we first investigated the microbial population in fresh milk, using a combination of cultivation-dependent and cultivation-independent methods, and also determined the impact of 6-day storage at 4°C. Samples were stored under anaerobic conditions as common domestic procedures for refrigerated storage at 4°C or freezing at − 20°C likely result in oxygenation of milk, depending on the handling and packaging. These conditions may hinder the delivery of natural mother’s milk microbes, especially strict anaerobes, which may play a role in the colonization of the neonate gut.

### Total Bacterial Load in Mature Human Breast Milk

The total viable bacteria counts in breast milk determined using plate counts were reported in the range from 1.2 to 5.5 log CFU/ml in breast milk of South African mothers (*n* = 55) (González et al., 2013), between log 2 and 6 in mothers own milk (*n* = 12) from preterm infants (Cacho et al., 2017) and <log 4.4 CFU/ml in milk from Swiss mothers (*n* = 7) (Jost et al., 2013). In agreement, our data confirmed that the number of bacterial cells recovered depended on the type of cultivation medium used and on culture conditions. In this study we applied the MPN method under strict anaerobic conditions and YCFA, a medium developed for the cultivation of gut microbes (Duncan et al., 2002). We supplied mucin or lactose, which are major carbohydrates available to breast milk and gut microbes, to the growth media, and also added acetate, propionate, and lactate in a ratio that mimicked metabolite profiles determined in infant feces (Pham et al., 2016). Bacterial cell counts determined in strict anaerobically prepared YCFA were higher in several samples than for a routinely used standard medium such as Columbia broth suggesting adapted YCFA broth as a suitable medium to recover a wide range of human milk microbes by cultivation.

Varying values were reported for the total bacterial load in human breast milk depending on the methodology applied. Quantification of 16S rRNA gene sequences suggested a bacterial load of up 10^6–7^ cells/ml of milk after correcting for multiple 16S rRNA gene copies per cell (Collado et al., 2009; Boix-Amorós et al., 2016), which is on average around log 1 cells/ml higher than tested with cultivation-based (González et al., 2013; Jost et al., 2013). This may be explained by incomplete recovery of all viable microbes by culture and/or by detection of 16S rRNA material from dead or lysed cells. During cultivation bacteria may grow directly on the sugar source present in the medium, or on intermediate metabolites produced by other microbes in cross-feeding. For e.g., only a minority of specialist microbes is adapted to degrade the complex mucin polymers and nevertheless we obtained high cell counts when mucin was provided as sole carbohydrate source. We also showed before that infant gut microbes can form trophic networks from mucin to yield SCFA (Schwab et al., 2017; Bunesova et al., 2018).

### Diversity of Breast Milk Microbes

Using a combined culture-dependent and -independent approach, we observed differently prevalent and abundant microbial subpopulations in the breast milk samples indicating high inter-individual variability of breast milk microbiota, akin to infant fecal microbiota (Pham et al., 2016). Our data are in agreement with previous culture-independent studies using 16S rRNA gene amplicon sequencing identifying a range of mainly members of the *Firmicutes* and *Proteobacteria* at proportions differing among individuals and studies (Boix-Amorós et al., 2016; Sakwinska et al., 2016; Urbaniak et al., 2016; Li et al., 2017; Simpson et al., 2017). We also attempted to determine microbiota composition using 16S rRNA amplicon sequencing. However, as similarly reported by Sakwinska et al. (2016), and Simpson et al. (2017), the first PCR failed to yield a quantifiable PCR product, likely the bacterial load in milk samples was at or below the limit of log 6 copies/ml (Biesbroek et al., 2012). Some samples were nevertheless sequenced, but did not yield sufficient reads. We then tested a nested PCR approach similar to Boix-Amorós et al. (2016) to first enrich the target amplicon, however, this approach yielded a high proportion (13–70%) of unclassified reads even at phylum likely due to low initial cell numbers and overamplification due to the nested PCR approach.

### Major Viable Bacterial Populations in Breast Milk Samples

To our surprise, we neither cultivated any *Bifidobacterium* nor amplified *Bifidobacterium* 16S rRNA genes using qPCR from fresh and stored breast milk samples while low abundant populations were detected in MPN samples using 16S rRNA gene sequencing. Previous studies reported the isolation of bifidobacteria (Martin et al., 2009; Solis et al., 2010; Jost et al., 2013; Kozak et al., 2015; Murphy et al., 2017). However, isolation frequency varied between 6 and 35% of the donors (Martin et al., 2009: 8/23; González et al., 2013: 6/55; Jost et al., 2013: 1/7; Murphy et al., 2017:1/10; Sakwinska et al., 2016: 5/90). *Bifidobacterium* were also not detected in breast milk when metagenomics was employed (Pärnänen et al., 2018), suggesting that *Bifidobacterium* spp. are a variable component of the human breast milk microbiota, or that they cannot be readily detected by the applied methods. *Bifidobacterium* spp. were suggested to be indicator organism providing evidence for the existence of an entero-mammary pathway as identical strains have been isolated from mother-infant pairs (Jost et al., 2014; Murphy et al., 2017).

Culture independent 16S rRNA gene analysis frequently (Jost et al., 2013; Boix-Amorós et al., 2016; Urbaniak et al., 2016; Li et al., 2017) but not always (Sakwinska et al., 2016; Simpson et al., 2017) suggests the presence of strict anaerobes of the *Clostridiales*, and *Bacteroidetes* in breast milk samples, however, isolates have not been obtained yet. In agreement, we also recovered 16S rRNA genes of *Clostridiales* and *Bacteroidetes* in fresh and stored milk. After anaerobic storage the prevalence of *Bacteroidetes* increased 3-fold while *Clostridium* clusters IV and XIVa of the *Clostridiales* increased in abundance or became detectable in seven samples, indicating that (1) viable strict anaerobes were present in breast milk albeit at low abundance, and that (2) anaerobic storage of breast milk at 4°C promoted the growth of some strict anaerobes.

(Unclassified) *Bacillaceae* were a major viable bacterial population recovered in MPN samples in agreement with *Firmicutes* being the major phylum present in breast milk according to qPCR analysis. The presence of *Bacillus* spp. in breast milk was observed before (Kumar et al., 2016; Urbaniak et al., 2016; Cacho et al., 2017; Simpson et al., 2017).

Using cultivation, we recovered *S. epidermis, Streptococcus* spp. and *C. acnes* from mMRS agar plates which belong to the natural skin microbiota (Grice et al., 2008). These species were already isolated by other researchers and are considered milk microbes (Collado et al., 2009; Hunt et al., 2011; Jost et al., 2013).

### Major HMO Composition Is Maintained After Storage Despite Bacterial Metabolic Activity

Lactose and HMO are major carbohydrates of breast milk constitute an important source of nutrients for the infant and the gut microbiota. Because of their resistance to enzymatic hydrolysis in the intestine, HMO represent the first prebiotic ingested (Engfer et al., 2000). Certain species of infant gut microbes such as *Bifidobacterium longum* subsp. *infantis* or *Bifidobacterium bifidum* are able to degrade and metabolize HMO (Garrido et al., 2015; Schwab et al., 2017); HMO also directly interact with pathogens (Bode, 2012; Kunz et al., 2014; Murphy et al., 2017). Our study shows that anaerobic cold storage of human milk for up to 6 days efficiently control both bacterial growth and degradation of HMO. HMO composition had been shown to vary among women, and depend on period of lactation and on the expression of specific glycosyltransferases in the mammary glands (Kunz et al., 2014) and also in this study the HMO profiles differed between donors. Lactose concentrations exhibited changes during storage, either decreasing or increasing depending on the milk sample. An apparent increase of lactose content during storage of human milk was also reported by Chang et al. (2012). Anaerobic cold storage of the milk did not significantly impact major HMO content and total bacterial load of human milk samples but led to changes in abundance of main bacterial groups. Milk does not contain oxygen until it is expressed from the mammary gland and comes in contact with air. Collection and home storage of milk may introduce oxygen in the milk, depending on the collection and storage container, which may affect the growth and viability of oxygen-sensitive bacteria.

### Butyrate Is a Component of Fresh Breast Milk

The majority of breast milk samples contained the SCFA butyrate which is in agreement with previous reports (Bruce German and Dillard, 2010; Aitoro et al., 2015). Human milk fat, which is mainly composed of triglycerides, contains less butyrate than cow milk (0.4 versus 2–3% by weight, respectively) (Bruce German and Dillard, 2010). Free butyrate concentrations remained stable during storage or even slightly increased due to degradation of lipids. The later may be due to the activity of milk lipases during cold storage (Freed et al., 1989) and possibly by the activity of butyrate producers despite being present at low abundance.

## Conclusion

Our results indicate the presence of viable strict anaerobes such as *Bacteroidetes* and *Clostridum* clusters IV and XIVa. YCFA supplemented with mucin or lactose was identified a suitable medium to recover a wide range of breast milk microbes by cultivation. Anaerobic cold storage of the milk, albeit at the moment not routinely used for preservation, did not significantly impact major HMO content and total bacterial load of human milk samples but led to changes in abundance of main bacterial groups.

## Data Availability Statement

The datasets generated for this study can be found in the ncbi’s sra archive, Bioproject PRJNA557136.

## Ethics Statement

The study involving human participants was reviewed and approved by the Ethics Committee of the ETH Zürich (2018-N-14). The participants provided their written informed consent to participate in this study.

## Author Contributions

MV, CL, and CS conceived the study. EV and AG conducted the experiments and run analysis. EV, AG, and CS analyzed the data. EV and CS wrote the manuscript with the support of all authors.

## Conflict of Interest

 MV was employed by the company Medela A/S Switzerland. The remaining authors declare that the research was conducted in the absence of any commercial or financial relationships that could be construed as a potential conflict of interest.

## Footnotes

1 https://www.fda.gov/food/laboratory-methods-food/bam-appendix-2-most-probable-number-serial-dilutions

## References

[B1] Ahrabi A. F. Handa D. Codipilly C. N. Shah S. Williams J. E. McGuire M. A. (2016). Effects of extended freezer storage on the integrity of human milk. *J. Ped.* 177 140–143. 10.1016/j.jpeds.2016.06.024 27423174

[B2] Aitoro R. Paparo L. Di Costanzo M. Nocerino R. Amoroso A. Amato F. (2015). Breast milk butyrate as protective factor against food allergy. *Dig. Dis. Liver Dis.* 47:e274. 10.3390/nu9070672 28657607 PMC5537787

[B3] Andriantsoanirina V. Allano S. Butel M. J. (2013). Tolerance of *Bifidobacterium* human isolates to bile, acid and oxygen. *Anaerobe* 21 39–42. 10.1016/j.anaerobe.2013.04.005 23598280

[B4] Ballard J. Morrow A. L. (2013). Human milk composition: nutrients and bioactive factors. *Ped. Clin. North Am.* 60 49–74. 10.1016/j.pcl.2012.10.002 PMC3586783 23178060

[B5] Bartosch S. Fite A. MacFarlane G. T. McMurdo M. E. (2004). Characterization of bacterial communities in feces from healthy elderly volunteers and hospitalized elderly patients by using real-time PCR and effects of antibiotic treatment on the fecal microbiota. *Appl. Environ. Microbiol.* 70 3575–3581. 15184159 10.1128/AEM.70.6.3575-3581.2004 PMC427772

[B6] Biesbroek G. Sanders E. A. M. Roeselers G. Wang X. Caspers M. P. M. Trzciński K. (2012). Deep sequencing analyses of low density microbial communities: working at the boundary of accurate microbiota detection. *PLoS One* 7:e32942. 10.1371/journal.pone.0032942 22412957 PMC3295791

[B7] Bode L. (2012). Human milk oligosaccharides: every baby needs a sugar mama. *Glycobiology* 22 1147–1162. 10.1093/glycob/cws074 22513036 PMC3406618

[B8] Boix-Amorós A. Collado M. C. Mira A. (2016). Relationship between milk microbiota, bacterial load, macronutrients, and human cells during lactation. *Front. Microbiol.* 7:492 10.3389/fmicb.2016.00492 PMC4837678 27148183

[B9] Bruce German J. Dillard C. J. (2010). Saturated fats – a perspective from lactation and milk composition. *Lipids* 45 915–923. 10.1007/s11745-010-3445-9 20652757 PMC2950926

[B10] Bunesova V. Lacroix C. Schwab C. (2018). Mucin-cross-feeding of infant bifidobacteria and *Eubacterium hallii*. *Microb. Ecol.* 75 228–238. 10.1007/s00248-017-1037-4 28721502

[B11] Cabrera-Rubio R. Collado C. M. Laitinen K. Salminen S. Isolauri E. Mira A. (2012). The human milk microbiome changes over lactation and is shaped by maternal weight and mode of delivery. *Am. J. Clin. Nutr.* 96 544–551. 10.3945/ajcn.112.037382 22836031

[B12] Cabrera-Rubio R. Mira-Pascual L. Collado M. C. (2016). Impact of mode of delivery on the milk microbiota composition of healthy women. *J. Dev. Origins Health Dis.* 7 54–60. 10.1017/S2040174415001397 26286040

[B13] Cacho N. T. Harrison N. A. Parker L. A. Padgett K. A. Lemas D. J. Marcial G. E. (2017). Personalization of the microbiota of donor human milk with mother’s own milk. *Front. Microbiol.* 8:1470. 10.3389/fmicb.2017.01470 28824595 PMC5541031

[B14] Chang Y.-C. Chen C.-H. Lin M.-C. (2012). The macronutrients in human milk change after storage in various containers. *Ped. Neonatol.* 53 205–209. 10.1016/j.pedneo.2012.04.009 22770111

[B15] Collado M. C. Delgado S. Maldonado A. Rodríguez J. M. (2009). Assessment of the bacterial diversity of breast milk of healthy women by quantitative real-time PCR. *Lett. Appl. Microbiol.* 48 523–528. 10.1111/j.1472-765X.2009.02567.x 19228290

[B16] Duncan S. H. Barcenilla A. Stewart C. S. Pryde S. E. Flint H. J. (2002). Acetate utilization and butyryl coenzyme a (CoA):acetate-CoA transferase in butyrate-producing bacteria from the human large intestine. *Appl. Environ. Microbiol.* 68 5186–5189. 12324374 10.1128/AEM.68.10.5186-5190.2002 PMC126392

[B17] Engfer M. B. Stahl B. Finke B. Sawatzki G. Daniel H. (2000). Human milk oligosaccharides are resistant to enzymatic hydrolysis in the upper gastrointestinal tract. *Am. J. Clin. Nutr.* 71 1589–1596. 10837303 10.1093/ajcn/71.6.1589

[B18] Fernández L. Langa S. Martín V. Maldonado A. Jiménez E. Martín R. (2013). The human milk microbiota: origin and potential roles in health and disease. *Pharmacol. Res.* 69 1–10. 10.1016/j.phrs.2012.09.001 22974824

[B19] Fleischer Michaelsen K. Skafte L. Badsberg J. H. Jorgensen M. (1990). Variation in macronutrients in human bank milk: influencing factors and implications for human milk banking. *J. Ped. Gastroenterol. Nutr.* 11 229–239. 2395063 10.1097/00005176-199008000-00013

[B20] Freed L. M. Berkow S. E. Hamosh P. York C. M. Mehta N. R. Hamosh M. (1989). Lipases in human milk: effect of gestational age and length of lactation on enzyme activity. *J. Am. Coll. Nutr.* 8 143–150. 2708730 10.1080/07315724.1989.10720289

[B21] Furet J. P. Firmesse O. Gourmelon M. Bridonneau C. Tap J. Mondot S. (2009). Comparative assessment of human and farm animal faecal microbiota using real-time quantitative PCR. *FEMS Microbiol. Ecol.* 68 351–362. 10.1111/j.1574-6941.2009.00671.x 19302550

[B22] Garrido D. Ruiz-Moyano S. Lemay D. G. Sela D. A. German J. B. Mills D. A. (2015). Comparative transcriptomics reveals key differences in the response to milk oligosaccharides of infant gut-associated bifidobacteria. *Sci. Rep.* 5:13517. 10.1038/srep13517 26337101 PMC4559671

[B23] González R. Mandomando I. Fumadó V. Sacoor C. Macete E. Alonso P. L. (2013). Breast milk and gut microbiota in African mothers and infants from an area of HIV prevalence. *PLoS One* 8:e80299. 10.1371/journal.pone.0080299 24303004 PMC3841168

[B24] Grice E. A. Kong H. H. Renaud G. Young A. C. Nisc Comparative Sequencing Program, Bouffard G. G. (2008). A diversity profile of the human skin microbiota. *Genome Res.* 18 1043–1050. 10.1101/gr.075549.107 18502944 PMC2493393

[B25] Gunther M. Camb M. D. Stanier J. E. (1949). Diurnal variation in the fat content of breast-milk. *Lancet* 254 235–237. 10.1016/s0140-6736(49)91242-8 18136281

[B26] Guo X. Xia X. Tang R. Zhou J. Zhao H. Wang K. (2008). Development of a real-time PCR method for *Firmicutes* and *Bacteroidetes* in faeces and its application to quantify intestinal population of obese and lean pigs. *Lett. Appl. Microbiol.* 47 367–373. 10.1111/j.1472-765X.2008.02408.x 19146523

[B27] Haiden N. Ziegler E. Z. (2016). Human milk banking. *Ann. Nutr. Metabol.* 69 8–15. 10.1159/000452821 28103607

[B28] Hall B. (1979). Uniformity of human milk. *Am. J. Clin. Nutr.* 32 304–312. 570353 10.1093/ajcn/32.2.304

[B29] Heikkila M. P. Saris P. E. J. (2003). Inhibition of *Staphylococcus aureus* by the commensal bacteria of human milk. *J. Appl. Microbiol.* 95 471–478. 12911694 10.1046/j.1365-2672.2003.02002.x

[B30] Hunt K. M. Foster J. A. Forney L. J. Schütte U. M. E. Beck D. L. Abdo Z. (2011). Characterization of the diversity and temporal stability of bacterial communities in human milk. *J. Appl. Microbiol.* 95 471–478. 10.1371/journal.pone.0021313 21695057 PMC3117882

[B31] Jeurink P. V. van Bergenhenegouwen J. Jiménez E. Knippels L. M. Fernández L. Garssen J. (2013). Human milk: a source of more life than we imagine. *Ben. Microbes* 4 17–30. 10.3920/BM2012.0040 23271066

[B32] Jost T. Lacroix C. Braegger C. Chassard C. (2013). Assessment of bacterial diversity in breast milk using culture-dependent and culture-independent approaches. *Brit. J. Nutr.* 110 1253–1262. 23507238 10.1017/S0007114513000597

[B33] Jost T. Lacroix C. Braegger C. P. Rochat F. Chassard C. (2014). Vertical mother-neonate transfer of maternal gut bacteria via breastfeeding. *Environ. Microbiol.* 16 2891–2904. 10.1111/1462-2920.12238 24033881

[B34] Khodayar-Pardo P. Mira-Pascual L. Collado M. Martínez-Costa C. (2014). Impact of lactation stage, gestational age and mode of delivery on breast milk microbiota. *J. Perinatol.* 34 599–605. 10.1038/jp.2014.47 24674981

[B35] Kostric M. Milger K. Krauss-Etschmann S. Engel M. Vestergaard G. Schloter M. (2018). Development of a stable lung microbiome in healthy neonatal mice. *Microb. Ecol.* 75 529–542. 10.1007/s00248-017-1068-x 28905200

[B36] Kozak K. Charbonneau D. Sanozky-Dawes R. Klaenhammer T. (2015). Characterization of bacterial isolates from the microbiota of mothers’ breast milk and their infants. *Gut Microb.* 6 341–351. 10.1080/19490976.2015.1103425 26727418 PMC4826109

[B37] Kuai L. Nair A. Polz M. F. (2001). Rapid and simple method for the most-probable-number estimation of arsenic-reducing bacteria. *Appl. Environ. Microbiol.* 67 3168–3173. 11425737 10.1128/AEM.67.7.3168-3173.2001 PMC92996

[B38] Kumar H. du Toit E. Kulkarni A. Aakko J. Linderborg K. M. Zhang Y. (2016). Distinct patterns in human milk microbiota and fatty acid profiles across specific geographic locations. *Front. Microbiol.* 7:1619. 27790209 10.3389/fmicb.2016.01619 PMC5061857

[B39] Kunz C. Kuntz S. Rudloff S. (2014). “Bioactivity of human milk oligosaccharides,” in *Food Oligosaccharides*, eds Moreno J. F. Sanz M. L. (Hoboken, NJ: Wiley), 1–20.

[B40] Kunz C. Rudloff S. Baier W. Klein N. Strobel S. (2000). Oligosaccharides in human milk: structural, functional, and metabolic aspects. *Ann. Rev. Nutr.* 20 699–722. 10940350 10.1146/annurev.nutr.20.1.699

[B41] Li S.-W. Watanabe K. Hsu C.-C. Chao S.-H. Yang Z.-H. Lin Y.-J. (2017). Bacterial composition and diversity in breast milk samples from mothers living in Taiwan and mainland China. *Front. Microbiol.* 8:965. 10.3389/fmicb.2017.00965 28611760 PMC5447776

[B42] Marín M. Arroya R. Jiménez E. Gómez A. Fernández L. Rodríguez J. M. (2009). cold storage of human milk: effect on its bacterial composition. *J. Pediatric Gastroenterol. Nutr.* 49 343–348. 10.1097/MPG.0b013e31818cf53d 19516191

[B43] Martin R. Jiménez E. Heilig H. Fernández L. Marín M. Zoetendal E. G. (2009). Isolation of bifidobacteria from breast milk and assessment of the bifidobacterial population by PCR-denaturing gradient gel electrophoresis and quantitative real-time PCR. *Appl. Environ. Microbiol.* 75 965–969. 10.1128/AEM.02063-08 19088308 PMC2643565

[B44] Martineau F. Picard F. J. Ke D. Paradis S. Roy P. H. Ouellette M. (2001). Development of a PCR assay for identification of staphylococci at genus and species levels. *J. Clin. Microbiol.* 39 2541–2547. 11427566 10.1128/JCM.39.7.2541-2547.2001 PMC88182

[B45] Matsuki T. Watanabe K. Fujimoto J. Takada T. Tanaka R. (2004). Use of 16S rRNA gene-targeted group-specific primers for real-time PCR analysis of predominant bacteria in human feces. *Appl. Environ. Microbiol.* 70 7220–7228. 15574920 10.1128/AEM.70.12.7220-7228.2004 PMC535136

[B46] Moossavi S. Sepehri S. Robertson B. Sears M. R. Khafipour E. Azad M. B. (2019). Composition and variation of the human milk microbiota are influenced by maternal and early-life factors. *Cell Host & Microb.* 25 324–335. 10.1016/j.chom.2019.01.011 30763539

[B47] Murphy K. Curley D. O’Callaghan T. F. O’Shea C.-A. Dempsey E. M. O’Toole P. W. (2017). The composition of human milk and infant faecal microbiota over the first three months of life: a pilot study. *Sci. Rep.* 7:40597. 10.1038/srep40597 28094284 PMC5240090

[B48] Newburg D. (2005). Innate immunity and human milk. *J. Nutr.* 135 1308–1312. 15867330 10.1093/jn/135.5.1308

[B49] Pärnänen K. Karkman A. Hultman J. Lyra C. Bengtsson-Palme J. Larsson D. G. J. (2018). Maternal gut and breast milk microbiota affect infant gut antibiotic resistome and mobile genetic elements. *Nat. Comm.* 9 3891. 10.1038/s41467-018-06393-w 30250208 PMC6155145

[B50] Petherick A. (2010). Development: mother’s milk: a rich opportunity. *Nature* 468 S5–S7. 21179083 10.1038/468S5a

[B51] Pham V. T. Lacroix C. L. Braegger C. P. Chassard C. (2016). Early colonization of functional groups of microbes in the infant gut. *Environ. Microbiol.* 18 2246–2258. 10.1111/1462-2920.13316 27059115

[B52] Price R. R. Viscount H. B. Stanley M. C. Leung K. P. (2007). Targeted profiling of oral bacteria in human saliva and *in vitro* biofilms with quantitative real-time PCR. *Biofouling* 23 203–213. 17653931 10.1080/08927010701251169

[B53] Ramirez-Farias C. Slezak K. Fuller Z. Duncan A. Holtrop G. Louis P. (2009). Effect of inulin on the human gut microbiota: stimulation of *Bifidobacterium adolescentis* and *Faecalibacterium prausnitzii*. *Br. J. Nutr.* 101 541–550. 10.1017/S0007114508019880 18590586

[B54] Rasmussen K. M. Geraghty S. R. (2011). The quiet revolution: breastfeeding transformed with the use of breast pumps. *Am. J. Public Health* 101 1356–1359. 10.2105/AJPH.2011.300136 21680919 PMC3134520

[B55] Rinttilä T. Kassinen A. Malinen E. Krogius L. Palva A. (2004). Development of an extensive set of 16S rDNA targeted primers for quantification of pathogenic and indigenous bacteria in faecal samples by real-time PCR. *J. Appl. Microbiol.* 97 1166–1177. 15546407 10.1111/j.1365-2672.2004.02409.x

[B56] Rocha Martin V. N. Schwab C. Krych L. Voney E. Geirnaert A. Braegger C. (2018). Colonization of *Cutibacterium avidum* during infant gut microbiota establishment. *FEMS Microbiol. Ecol.* 95:fiy215. 10.1093/femsec/fiy215 30388209

[B57] Sakwinska O. Moine D. Delley M. Combremont S. Rezzonico E. Descombes P. (2016). Microbiota in breast milk of Chinese lactating mothers. *PLoS One* 11:e0160856. 10.1371/journal.pone.0160856 27529821 PMC4987007

[B58] Schwab C. Ruscheweyh H. J. Bunesova V. Pham V. T. Beerenwinkel N. Lacroix C. (2017). Trophic interactions of infant bifidobacteria and *Eubacterium hallii* during L-fucose and fucosyllactose degradation. *Front. Microbiol.* 8:1–14. 10.3389/fmicb.2017.00095 28194144 PMC5277004

[B59] Simpson M. R. Avershina E. Storrø O. Johnsen R. Rudi K. Øien T. (2017). Breastfeeding-associated microbiota in human milk following supplementation with *Lactobacillus rhamnosus* GG, *Lactobacillus acidophilus* La-5, and *Bifidobacterium animalis* subspecies lactis Bb-12. *J. Dairy Sci.* 101 1–11. 10.3168/jds.2017-13411 29248229

[B60] Solis G. de los Reyes-Gavilan C. G. Fernández N. Margolles A. Gueimonde M. (2010). Establishment and development of lactic acid bacteria and bifidobacteria microbiota in breast-milk and the infant gut. *Anaerobe* 166 307–310. 10.1016/j.anaerobe.2010.02.004 20176122

[B61] Stoddard S. F. Smith B. J. Hein R. Roller B. R. K. Schmidt T. M. (2015). rrnDB: improved tools for interpreting rRNA gene abundance in bacteria and archaea and a new foundation for future development. *Nucleic Acid Res.* 43 D593–D598. 10.1093/nar/gku1201 25414355 PMC4383981

[B62] Stolz P. Böcker G. Hammes W. P. Vogel R. F. (1995). Utilization of electron acceptors by lactobacilli isolated from sourdough. *Z. Lebensm. Unters. Forsch.* 201 91–96.

[B63] Sutton S. (2010). The most probable number method and its uses in enumeration, qualification, and validation. *J. Validation Technol.* 16 35–38.

[B64] Urbaniak C. Angelini M. Gloor G. B. Reid G. (2016). Human milk microbiota profiles in relation to birthing method, gestation and infant gender. *Microbiome* 4:1. 10.1186/s40168-015-0145-y 26739322 PMC4702315

[B65] Vlková E. Salmonová H. Bunešová V. Geigerová M. Rada V. Musilová S. (2015). A new medium containing mupirocin, acetic acid, and norfloxacin for the selective cultivation of bifidobacteria. *Anaerobe* 34 27–33. 10.1016/j.anaerobe.2015.04.001 25865525

[B66] World Health Organization (2001). *The Optimal Duration of Exclusive Breastfeeding - Report of an Expert Consultation. Department of Nutrition for Health and Development Department of Child and Adolescent Health and Development.* Geneva: World Health Organization.

[B67] World Health Organization (2003). *Global Strategy for Infant and Young Child Feeding.* Geneva: World Health Organization.

[B68] Yu G. Fadrosh D. Goedert J. J. Ravel J. Goldstein A. M. (2015). Nested PCR biases in interpreting microbial community structure in 16S rRNA gene sequence datasets. *PLoS One* 10:e0132253. 10.1371/journal.pone.0132253 26196512 PMC4509648

[B69] Yuhas R. Pramuk K. Lien E. L. (2006). Human milk fatty acid composition from nine countries varies most in DHA. *Lipids* 41 851–858. 17152922 10.1007/s11745-006-5040-7

