# Supplementary material for: Characterization of the Cultivable Microbiota in Fresh and Stored Mature Human Breast Milk
Source: Front Microbiol. 2019 Nov 20;10:2666. doi: 10.3389/fmicb.2019.02666 (PMC6879428; doi:10.3389/fmicb.2019.02666)
Supplement: Supplementary file 1 [file Data_Sheet_1.docx]

**Supplementary material**

**Characterization of the cultivable microbiota in fresh and stored mature human breast milk**

Clarissa Schwab^1^*, Evelyn Voney^1^, Alejandro Ramirez^1^, GarciaMichaela Vischer^2^, Christophe Lacroix^1^*

^1^Laboratory of Food Biotechnology, Department of Health Sciences and Technology, ETH Zürich, Zürich, Switzerland

^2^Medela AG, Baar, Switzerland

*** Correspondence:**clarissa.schwab@hest.ethz.ch

christophe.lacroix@hest.ethz.ch

**Keywords:**

Human breast milk, anaerobes, cultivation, storage

**Table S1.** Composition of YCFA media. YCFA was prepared without carbohydrate source (YCFAwo), with lactose (YCFA+Lact), or with mucin (YCFA+Muc)

| Ratio of added Component | Supplier |
| --- | --- |
| 1% (w/v) amicase | Sigma Aldrich, Switzerland |
| 0.25% (w/v) yeast extract | VWR International, Switzerland |
| 0.5% (w/v) sodium bicarbonate | Sigma Aldrich, Switzerland |
| 1.8% (w/v) lactose (replaced with 0.2% mucin or without addition of sugar) | Sigma Aldrich, Switzerland |
| 0.1% (v/v) vitamin solution [0.01% (w/v) biotin, 0.01% (w/v) cobalamin, 0.03% (w/v) p-aminobenzoic acid, 0.05% (w/v) folic acid, 0.15% (w/v) pyridoxamine] | Sigma Aldrich, Switzerland  VWR International, Switzerland |
| 0.31% (v/v) volatile fatty acid mix for the determination of cultivable cells [69.99% (v/v) acetic acid, 22.50% (v/v) lactic acid, 4.98% (v/v) propionic acid] | Sigma Aldrich, Switzerland |
| 0.02 % hemin [0.5 mg/ml] | Sigma Aldrich, Switzerland |
| 0.1% resazurin [1 mg/ml] | Sigma Aldrich, Switzerland |
| 0.1% (w/v) L-cysteine HCI | Sigma Aldrich, Switzerland |

**Table S2.** DNA concentrations detected in fresh and stored breast milk samples determined with QuBit.

| **Sample ID** | **[ng/µl]** |
| --- | --- |
| **2_0d** | 1.95 |
| **2_6d** | 1.32 |
| **3b_0d** | 0.18 |
| **3b_6d** | 0.35 |
| **4_0d** | 1.10 |
| **4_6d** | 1.29 |
| **5_0d** | 0.26 |
| **5_6d** | 0.37 |
| **6_0d** | 0.09 |
| **6_6d** | 0.13 |
| **7_0d** | 0.36 |
| **7_6d** | 1.55 |
| **8_0d** | 0.30 |
| **8_6d** | 0.27 |
| **9_0d** | 0.17 |
| **9_6d** | 0.47 |
| **10_0d** | 0.14 |
| **10_6d** | 0.61 |
| **11_0d** | 0.38 |
| **11_6d** | 0.19 |
| **12_0d** | 0.88 |
| **12_6d** | 1.09 |
| **13_0d** | 0.06 |
| **13_6d** | 0.09 |
| **14_0d** | 34.50 |
| **14_6d** | 39.30 |
| **15_0d** | 0.35 |
| **15_6d** | 0.27 |
| **16_0d** | 0.35 |
| **16_6d** | 0.81 |
| **17_0d** | 0.09 |
| **17_6d** | < 0.50 ng/ml |
| **18_0d** | 2.30 |
| **18_6d** | 0.72 |
| **19_0d** | 0.40 |
| **19_6d** | 0.18 |
| **20_0d** | < 0.50 ng/ml |
| **20_6d** | < 0.50 ng/ml |
| **21_0d** | 7.88 |
| **21_6d** | 4.35 |
| **22_0d** | 2.44 |
| **22_6d** | 1.11 |

**Table S3. Prevalence and mean abundance of selected bacterial groups in fresh and stored breast milk.** Mean abundance of bacterial groups in fresh and stored milk samples (n=19) was calculated from positive samples only.

| **Bacterial group** | **Fresh milk** | | **Stored milk** | |
| --- | --- | --- | --- | --- |
|  | Prevalence | Mean abundance (log cells/ml) | Prevalence | Mean abundance (log cells/ml) |
| Total bacteria | 19/19 | 5.4±0.5^A^ | 19/19 | 5.4±0.4^A^ |
| *Firmicutes* | 18/19 | 4.8±1.4^A^ | 19/19 | 5.2±0.4^A^ |
| *Lactobacillus*/ *Leuconostoc*/  *Pediococcus* spp. | 12/19 | 2.4±0.3^A^ | 14/19 | 2.5±0.4^A^ |
| *Staphylococcus* spp. | 5/19 | 1.5±0.3^A^ | 3/19 | 0.8±0.6^B^ |
| *Streptococcus* spp. | 15/19 | 2.7±0.3^A^ | 18/19 | 2.6±0.3^A^ |
| *Veillonella* spp. | 10/19 | 2.7±0.3^A^ | 10/19 | 2.6±0.3^A^ |
| *Clostridium* cluster IV | 6/19 | 2.3±0.4^A^ | 5/19 | 3.1±0.6^B^ |
| *Clostridium* cluster XIV | 5/19 | 2.7±0.2^A^ | 5/19 | 2.8±0.3^A^ |
| *Bacteroides/Prevotella/*  *Porphyromonas* | 3/19 | 2.8±0.3^A^ | 9/19 | 2.9±0.5^A^ |
| *Enterobacteriacaea* | 13/19 | 0.8±0.2^A^ | 11/19 | 1.1±0.3^A^ |
| *Bifidobacterium* spp. | nd^1^ | - | nd | - |
| *Propionibacterium/*  *Cutibacterium* spp. | 1/19 | - | 3/19 | - |

^1^not detected

^A^different letters indicate a significant difference between fresh and stored mean cell counts

**
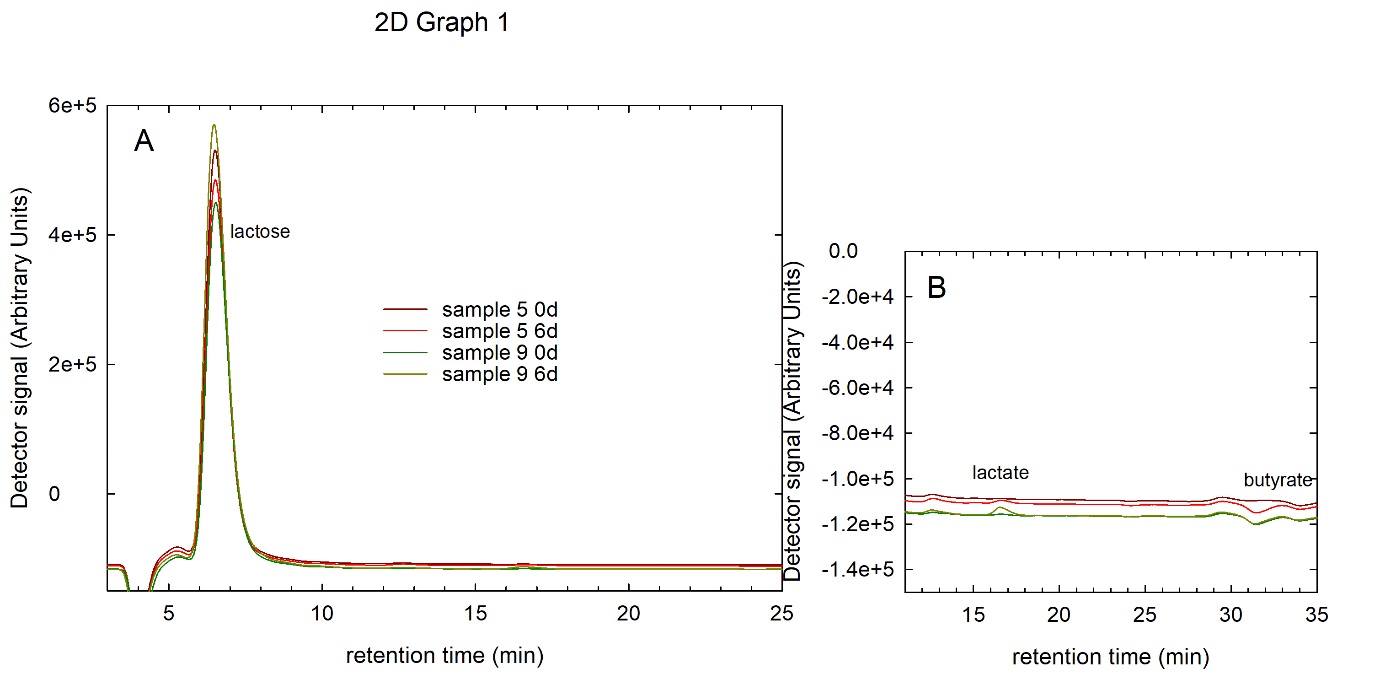
Figure S1. Representative chromatograms from determination of lactose, lactate and SCFA.** Lactose, lactate and SCFA concentrations were determined using HPLC-RI and external standards as references. Chromatograms from two samples before and after storage are shown.

**Figure S2.** **Viable cell counts in YCFA-based media before and after storage.** Boxes indicate the 25th and 75th percentiles. Whiskers indicate 10th and 90th percentiles, 5^th^ and 95^th^ percentiles are shown as dots. The dotted and solid lines indicate mean and median, respectively. Viable cell counts of individual samples and changes in abundance during storage are indicated by black dots and connecting lines.

**Figure S3. Relative abundance of bacterial phyla in selected fresh and stored breast milk samples.** Bacterial diversity was determined using 16S rRNA gene amplicon sequencing employing a nested PCR approach due to low bacterial load of the samples.
